# Post-Cardiac Arrest: Mechanisms, Management, and Future Perspectives

**DOI:** 10.3390/jcm12010259

**Published:** 2022-12-29

**Authors:** Taline Lazzarin, Carolina Rodrigues Tonon, Danilo Martins, Edson Luiz Fávero, Thiago Dias Baumgratz, Filipe Welson Leal Pereira, Victor Rocha Pinheiro, Raquel Simões Ballarin, Diego Aparecido Rios Queiroz, Paula Schmidt Azevedo, Bertha Furlan Polegato, Marina Politi Okoshi, Leonardo Zornoff, Sergio Alberto Rupp de Paiva, Marcos Ferreira Minicucci

**Affiliations:** Internal Medicine Department, Botucatu Medical School, Universidade Estadual Paulista (UNESP), Botucatu 18607-741, Brazil

**Keywords:** cardiac arrest, targeted temperature management, rehabilitation, physiopathology

## Abstract

Cardiac arrest is an important public health issue, with a survival rate of approximately 15 to 22%. A great proportion of these deaths occur after resuscitation due to post-cardiac arrest syndrome, which is characterized by the ischemia-reperfusion injury that affects the role body. Understanding physiopathology is mandatory to discover new treatment strategies and obtain better results. Besides improvements in cardiopulmonary resuscitation maneuvers, the great increase in survival rates observed in recent decades is due to new approaches to post-cardiac arrest care. In this review, we will discuss physiopathology, etiologies, and post-resuscitation care, emphasizing targeted temperature management, early coronary angiography, and rehabilitation.

## 1. Introduction

Cardiac arrest (CA) is an important public health issue (1). Survival of patients with out-of-hospital cardiac arrest (OHCA) is less than 15% (2), while that after in-hospital cardiac arrest (IHCA) is approximately 22% (3). A great proportion of these deaths occur after resuscitation due to post-cardiac arrest syndrome (PCAS). PCAS is a complex occurrence that is characterized by the ischemia-reperfusion injury that affects the whole body. Understanding physiopathology and discovering treatment strategies are essential to obtain better results. Despite advances in cardiopulmonary resuscitation (CPR) maneuvers, post-resuscitation care, which is started immediately after the sustained return of spontaneous circulation (ROSC), is of fundamental importance [1,2,3].

Thus, in this article, we will review PCAS, including the physiopathology, etiologies, and post-resuscitation care, emphasizing temperature management, early coronary angiography, and rehabilitation.

## 2. Physiopathology

PCAS is a complex and critical issue that was first described in the 1950s [4]. Understanding the pathophysiology of the ischemia-reperfusion (IR) injury that occurs in PCAS may elucidate therapeutic targets. We will describe systemic involvement in general, and later, we will highlight the particularities of specific organs.

### 2.1. Systemic Process of Ischemia and Reperfusion

Ischemia-reperfusion is characterized by blood supply restriction followed by restoration [5]. Ischemia reduces oxygen and nutrient supply, which is initially compensated for by a reduction in the systemic metabolic rate [6]; however, a sustained ischemic process leads to cell damage.

Oxygen deprivation impairs mitochondrial oxidative phosphorylation, shifting energy production to anaerobic metabolism, which generates tissue acidosis. Acid–base imbalance is responsible for several cellular dysfunctions. Energy stores are finite; once they are depleted, there is a failure of active cellular processes, such as the regulation of membrane ion pumps, resulting in electrolyte imbalance and, for example, sodium and water accumulation. These processes cause cell edema, calcium influx (which acts as a second messenger of several injury cascades via mitochondrial damage), changes in gene expression, and increased production of reactive oxygen species [7,8,9,10].

Tissue injury triggers the activation of inflammatory cascades. This activation can be beneficial by promoting tissue repair, or it can be harmful by triggering uncontrolled inflammation, perpetuating tissue damage. When inflammatory activation is harmful, it has a similar profile to that observed in sepsis, which is characterized by a high concentration of cytokines, although it usually occurs in a sterile environment [11]. A study on the plasma of patients after CA showed that it induced more in vitro endothelial cell death compared with plasma of septic shock patients, reinforcing its toxic character [12].

Inflammatory activation occurs in the ischemic period and during reperfusion. The mechanism for this involves innate and adaptative immune systems and the complement system. The innate system is activated by endogenous molecules called damage-associated molecular patterns, which are generated or released during cell injury [13,14]. Especially during the initial phase of reperfusion, innate immune cells are preponderant in inflammatory infiltrates [5]. The adaptative immune system is responsible for the activation of T lymphocytes and their products, which can cause tissue damage [15,16,17,18]. Humoral activation also contributes to further tissue damage. Finally, the complement system acts by differentiating healthy tissue from cellular debris and apoptotic and intruding cells [19]; it is activated by locally amplifying the inflammation. A detailed description of the inflammatory pathways activated at each moment of ischemia-reperfusion syndrome (IRS) is beyond the scope of this review, but we would like to emphasize the importance of inflammation.

Another IRS component is endothelial dysfunction. Endothelial tissues are among the most vulnerable to IR-induced injury [20]; damage thereof causes increased vascular permeability, hypercoagulability, vasoconstriction, and local inflammation [21,22]. The main stimulus for increased vascular permeability is the hypoxemia which occurs in the ischemic period [23]. Hypercoagulability occurs by platelet activation and by endogenous pro- and anticoagulant factors that result in an imbalance that can culminate in disseminated intravascular coagulation [24]. Regarding vasomotor tone, vasoconstricting substances predominate independently of smooth muscle function [25]. Vasoconstriction endothelial mechanisms are described in fully denervated transplanted hearts [26]. Additionally, endothelial dysfunction stimulates an inflammatory response including leukocyte recruitment, complement activation, and pro-inflammatory gene expression [27].

Regarding reperfusion injury, blood flow restoration is essential but deleterious. The supply of oxygen promotes the generation of free radicals (FR) [28], highly reactive molecules which have an unpaired electron and interact with nearby structures to achieve electrical stability. The most well-known examples include superoxide anions, hydrogen peroxide, and hydroxyl radicals. They are produced by neutrophils, eosinophils, and endothelial cells in various cellular processes [29]. FR can cause peroxidation of membrane fatty acids, enzyme inactivation, deoxyribonucleic acid (DNA) modifications, activation of inflammatory messengers, platelet induction, nitric oxide inactivation, and the release of vasoconstrictor agents, among other things. FR-induced lesions are called oxidative stress [28,30]. Under normal conditions, endogenous protective mechanisms (antioxidant enzymes, superoxide dismutase, catalase, and glutathione peroxidase) and FR scavengers (glutathione, α-tocopherol, and β-carotene) control oxidative damage [31]. However, in IRS, FR production overcomes the protection mechanisms, as already demonstrated in PCA patients [32].

In summary, the pathological processes we described above, i.e., hypoxemia, endothelial injury, platelet activation, inflammation, and reperfusion-induced injury, are closely related to each other, ultimately culminating in cell death. Initially, there were only two known pathways of cell death: apoptosis and necrosis. Necrosis is defined as uncontrolled cell death that damages adjacent structures by releasing cell contents and inflammation, while apoptosis represents a controlled form of cell death with minimal effect on the surrounding tissue and without leakage of cell contents, conferring a silent and anti-inflammatory character [33]. However, as studies have progressed, other pathways of cell death have been identified, such as autophagy, necroptosis, ferroptosis, and pyroptosis [34]. Each of these mechanisms is activated by distinct signaling pathways, culminating in different modes of cell death and different consequences for the organism [35].

Regardless of the predominant pathway, the classical mechanisms of injury that result in cell death include reduced adenosine triphosphate (ATP) synthesis [36], irreversible mitochondrial injury, and alteration of calcium homeostasis [37]. These processes result in the activation of enzymes such as phospholipases (causing damage to the membrane), proteases (which degrade the membrane and cytoskeleton), ATPases (which degrade ATP), and endonucleases (which degrade DNA), as well as oxidative stress and the loss of genome integrity [34].

### 2.2. Brain Injury

Neurological outcome is one of the main determinants of PCA survival, representing an important cause of mortality and morbidity [38]. Brain injury can be catastrophic. The central nervous system (CNS) does not have its own metabolic stores and is highly dependent on oxygen, being responsible for 20% to 25% of total body oxygen consumption [4].

For didactic purposes, we can divide brain injury into primary, which occurs immediately after blood flow cessation, and secondary, which occurs after the return of circulation [39]. In primary injury, there is a deficit of supplements, calcium dyshomeostasis [37], mitochondrial dysfunction [40,41], oxidative stress [42,43], inflammatory activation [44,45,46], and excitotoxicity. Secondary injury is caused by microvascular dysfunction, cerebral edema, oxygen and carbon dioxide concentrations, hyperthermia, anemia, hyperglycemia, and seizures.

Microvascular dysfunction results from microthrombi, vasoconstriction, and disruption of the blood–brain barrier, resulting in increased vascular resistance, reduced blood flow, and edema. Cerebral edema is vasogenic and cytotoxic. The former is mainly mediated by aquaporins which cause fluid displacement in the interstitium. Cytotoxicity occurs due to energy depletion and dysregulation of membrane ion pumps, leading to intracellular sodium and water retention. Regardless of the mechanism, edema occurs in a fixed volume system and, therefore, the volumetric increase in the parenchyma causes intracranial hypertension, reduced perfusion, and even cerebral herniation [47].

Hypoxemia is deleterious to neuronal function, but hyperoxia is also harmful because FR production increases. Thus, it is important to maintain strict control of oxygen concentrations [48]. Carbon dioxide modulates vasomotor tone, interfering with blood flow and intracranial pressure. Hypercapnia and hypocapnia induce, respectively, vasodilation and vasoconstriction. Anemia reduces arterial oxygen content, contributing to ischemia. The arterial oxygen content is primarily dependent on hemoglobin [49]. Hyperthermia increases metabolic oxygen demand, reduces seizure threshold, and induces apoptosis, causing cell death. Hyperglycemia is associated with poor neurologic outcomes after CA, and studies have linked glycemic control with PCA survival [50,51]. Seizures are associated with a worse neurological prognosis and death. This manifestation is a cause and a consequence of PCA brain injury and increases brain metabolism by up to three times [52].

Another particularity of brain tissue is its limited tolerance of ischemia. The oxygen deprivation time required for the onset of cellular damage in the CNS is shorter than in other tissues [53,54]. Despite the early onset of brain injury, evidence has shown an increase in injury cascades up to 7 days after reperfusion, probably due to secondary injury mechanisms, providing a wide therapeutic window for neuroprotective strategies after CA [55,56,57,58].

### 2.3. Myocardial Injury

Post-ischemic myocardial dysfunction was first described in the 1970s [59], and in 1982, it was consolidated as a clinical entity by Braunwald and Kloner [60]. The incidence of myocardial dysfunction can reach 68%, usually causing early and intense dysfunction that can be completely reversed after 48–72 h [61,62].

Cardiac dysfunction affects systole and diastole. Systolic deficit is demonstrated by reduced global contractility, cardiac index, and ejection fraction [63]. Studies have shown a difference of up to 14% in the ejection fraction of patients with and without myocardial dysfunction after CA [62]. Diastolic deficit occurs as an extension of ischemic contracture by uncontrolled activation of the contractile machinery, increased rigidity, and decreased myocardial compliance [64]. The severity of ischemic contracture is proportional to the duration of ischemia and is maximal during the metabolic phase (after about 10 min) of CA [65]. The repercussions include ventricular wall thickening, deficient relaxation, and reduced end-diastolic volume [64,66]. A common pathway of systolic–diastolic injury that is worth mentioning is cardiac edema; this occurs due to reduced lymphatic flow, a consequence of the loss of rhythmic contraction, and to increased microvascular permeability. Studies indicate that a 3.5% gain in myocardial water results in a 30–50% decline in cardiac output [67].

Another myocardial particularity is metabolic and electrical status. The heart’s main energy source is the oxidation of fatty acids, providing 60–70% of its energy needs [68]. However, after critical ischemia, glucose dominates as the energy source because lipid oxidation, despite being more effective, consumes more oxygen. As a consequence of this metabolic deviation, there is less ATP production and potentially an accumulation of toxic lipid substances, causing myocyte apoptosis, myocardial fibrosis, and, ultimately, cardiac dysfunction [69]. In contrast, when reperfusion takes place, lipids once again become the main energy source, and oxygen consumption increases, also leading to cardiac dysfunction [70]. Regarding cardiac electrical potential, IRS can interfere with myocardial electrical control. Energy depletion, ionic imbalance, and the presence of reactive oxygen species destabilize cardiac electrical activity, causing membrane depolarization, shortening the potential of action, and stimulating arrhythmic activity [64,71]. Ventricular premature beats, ventricular tachycardia, and episodes of fibrillation may occur, especially in the first 5 to 20 min after ROSC [72].

CPR can also contribute to myocardial injury, initially directly due to chest compressions [73], but also due to exogenous factors such as the administration of epinephrine, which, by β-adrenergic stimulation, increases oxygen consumption and the probability of arrhythmias [74], while electric shocks produce cell injury proportional to the amount of energy used [75]. In addition, CPR can also cause reperfusion injuries, because the coronary blood flow during resuscitation is low and does not maintain aerobic myocardial metabolism; however, it is sufficient to promote the deleterious effects of reperfusion [76].

### 2.4. Other Organs

Respiratory dysfunction occurs in up to 50% of patients. It may be caused by pulmonary edema, contusion, or atelectasis. Persistent vasoconstriction causes loss of self-regulation of blood flow to the kidneys, reducing glomerular filtration and promoting a pro-inflammatory state due to endothelial damage that can perpetuate the mechanisms of renal injury [77,78]. Gastrointestinal tract (GIT) insult is often underestimated due to the difficulty of assessment. However, in addition to being a victim of circulatory failure, the GIT perpetuates the systemic inflammatory response, because a loss of barrier integrity favors the systemic translocation of endotoxins [6]. GIT injury occurs especially in the reperfusion period. Injuries induced by three hours of ischemia followed by one hour of reperfusion are more severe than those induced by four hours of ischemia alone [79]. The liver is a resistant organ with unique protection mechanisms against ischemia, such as double irrigation (portal vein and hepatic artery), high permeability of the hepatic sinusoids (favoring diffusion and allowing an increase of up to 90% in the extraction of available oxygen [80,81]), and great glycolytic capacity to generate ATP in the absence of oxygen. Even so, liver injury is still observed in around 24% of cases and is strongly associated with mortality and worse neurological outcome in victims of CA [82]. Finally, adrenal dysfunction due to direct glandular damage to the hypothalamic–pituitary–adrenal axis can reduce the release of catecholamines and corticosteroids [83].

## 3. Investigating the Etiology of Cardiac Arrest

Identifying the cause of CA is important for management after ROSC and may improve outcomes in CA victims [84,85]. Nevertheless, this can be challenging, even for experienced clinicians and ER personnel. The patient can be in a comatose state after resuscitation, making it impossible to obtain a direct history. Even those who achieve ROSC with a conscious status may have retrograde amnesia [86] and may report nonspecific symptoms after resuscitation. Chest pain after resuscitation, for example, may be due to acute coronary syndrome (ACS) or to thoracic compressions.

Because of the difficulty in obtaining a history of the patient, it is fundamental to obtain information from family and witnesses. Physical examination may suggest some etiologies of CA [86]. Furthermore, some clinical findings have prognostic value. An observational single-center study showed that a Glasgow Coma Scale (GCS) score less than or equal to 8 in patients with ROSC after OHCA was associated with only 9% survival, whereas a value over 8 was associated with a survival rate of 94% [87]. Table 1 summarize these findings.

There are many potential etiologies of CA, which can be grouped into cardiac and non-cardiac causes [88]; the majority are cardiac, i.e., between 50–87% [88,89,90,91], although some studies may overestimate the percentage [92]. Respiratory etiologies are the main non-cardiac cause, occurring in about 11–40% of cases [88,89,90]. Other etiologies include metabolic, traumatic, and neurologic [93,94,95,96,97], as shown in Table 2.

To assess the diagnosis of CA, the first exam that should be performed after ROSC is an electrocardiogram (ECG) [3,11]. The presence of an ST-segment elevation (STE) accurately identifies acute coronary lesions and cardiac causes [95]. However, this technique may not be so sensitive, and the absence of STE does not exclude the possibility of an acute coronary lesion. Even the presence of STE may be a false positive. An observational study revealed that the earlier the ECG is performed after ROSC, the higher the chance of the STE being a false positive [96], which suggests that an ECG with STE should be repeated after initial evaluation. Despite the findings of the ECG, if a cardiac cause is suspected, the patient should undergo coronary angiography.

If a cardiac etiology is not the likely cause or if it was not identified by coronary angiography, other imaging exams should be performed. A systematic review analyzed the utility of noninvasive imaging exams performed after ROSC in patients with OHCA [98]. Despite their great utility, Petek et al. (insert date) concluded that more prospective studies are needed to better understand the impact of imaging exams on etiologic diagnoses of CA.

Point-of-care ultrasound in cardiac arrest (POCUS-CA) is an emerging tool that can help in CA evaluations. Even during cardiopulmonary resuscitation maneuvers, it can be useful in identifying possible causes of CA. Some protocols, for example, CASA [99] and SESAME [100], can identify some reversible causes, like pneumothorax and cardiac tamponade without prolonging CPR pulse checks [101]. After ROSC, POCUS-CA has some advantages in comparison with Computerized Tomography; for example, it can be used with unstable patients. Evidence has shown that POCUS realized within one hour after ROSC can provide a possible diagnosis in about 22% of the patients [102]. Due to the growing importance of ultrasound as a predictive tool, studies have highlighted the importance of strengthening the available evidence through high-quality studies to allow the integration of POCUS-CA into universal CPR algorithms [102].

Recently, some prospective studies have assessed the diagnostic value of whole-body computerized tomography (CT) after ROSC [103,104]. The results showed that an early whole-body CT identified a cause of CA in 39% of cases [103] and that it can be safely performed in patients without an obvious etiology. The European Society of Intensive Care Medicine recently published post-resuscitation guidelines with the recommendation of a brain CT and/or a contrasted pulmonary CT to diagnose non-cardiac causes of CA [105].

## 4. Post-Resuscitation Shock

Described in the 1980s by Negovsky, post-resuscitation shock occurs in 50–70% of patients with CA [106,107]. Hospital mortality attributable to this phenomenon usually occurs rapidly, representing around 20–55% of in-hospital deaths [38,108,109]. Post-resuscitation shock is a consequence of multiple organ dysfunction, such as myocardial ischemia [62], renal failure [110], ischemic hepatitis [111,112], and metabolic acidosis [113]. Factors related to this shock include the amount of epinephrine administered during resuscitation [61], male gender, shockable rhythm, and time to obtain ROSC [108].

The pathophysiology of post-resuscitation shock is the combination of IRS factors, such as myocardial dysfunction, vasoplegia, and hypovolemia, in addition to specific contributors, depending on the etiology of CA. Post-arrest myocardial dysfunction shares characteristics with cardiomyopathy post-cardiopulmonary bypass and is stress-induced and septic [114,115,116]. Myocardial dysfunction is early and severe. The ejection fraction transiently drops to values close to 40%, indicating a stunned myocardium [117]. Vasoplegia occurs due to a systemic inflammatory response mediated by cytokines and endothelial dysfunction [118,119,120], similar to the pathophysiology of sepsis. In addition, authentic sepsis can contribute to this hemodynamic profile, since infectious complications are common at this stage [120]. Other possible contributors include endogenous cortisol deficit due to relative adrenal insufficiency, present in up to 52% of these patients [121], and impaired hypothalamic vasopressin release [122]. Finally, hypovolemia occurs mainly due to vasoplegia and capillary leakage [123].

Therefore, in general, patients initially develop a low-output shock state secondary to cardiomyopathy, followed by severe vasodilation as systemic inflammation develops [61]. However, it is important to highlight that there is interindividual variability in the different mechanisms described, although all are closely related and result in a self-perpetuating vicious circle.

## 5. Post-Cardiac Arrest Care

**Figure 1:**
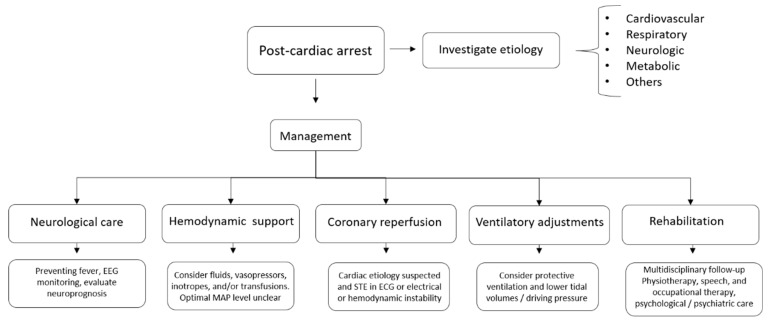
Post-cardiac arrest management.

The management of PCAS employs general intensive care strategies in addition to investigation and specific therapy regarding the precipitating cause. Here, we will describe, in general terms, our proposed approach for the treatment of post-arrest patients, as well as the perspectives for the future care of these patients (Figure 1).

### 5.1. Neurologic Care

Due to the importance of the CNS and susceptibility to sequelae, there is much interest in providing the best support for this system. In this regard, the first measure is to minimize secondary brain injury [124]. Several precipitants can exacerbate the damage caused by IRS, such as cerebral edema, oxygen and carbon dioxide concentrations, dysglycemias, anemia, seizures, and hyperthermia. Cerebral edema in clinical practice, despite the theoretical rationale, is rarely associated with significant increases in intracranial pressure [41,125,126,127], and therefore, no specific measures are recommended.

Regarding blood gas parameters, it is recommended to avoid hypocarbia or hypercarbia [128] and hypoxia or hyperoxia [129]. Regarding glycemic control, there is no specific target range, so it is recommended that such measures be performed as they would for other critically ill patients, using insulin to maintain blood glucose at 150 to 180 mg/dL [130]. Anemia and seizures should be monitored, and therapy should be implemented if necessary. Hyperthermia, given its importance and broad historical discussion, will be discussed as a specific topic.

Despite the knowledge gained regarding the mechanisms involved in neurological injury, no drug has been approved for neuroprotection after CA. Several therapies, such as barbiturates, glutamatergic antagonists, calcium channel blockers, antioxidants, and erythropoietin, among others, have failed to demonstrate benefits in humans. The lack of data in this field may be a result of overestimating preclinical evidence, as well as of differences between animal models and real patients. However, it is also worth noting that clinical trials, in general, lack comprehensive neuropsychological assessments because the main neurological outcome assessment scales, such as Cerebral Performance Categories or the Glasgow Outcome Scale, may not detect subtle changes in cognitive, behavioral, or functional recovery. Despite this, these instruments should be more widely used in routine clinical practice.

#### 5.1.1. Active Temperature Management

Active temperature management (ATM) is proposed for neuroprotection in the PCA period. Common cooling methods include endovascular cooling solution infusion and the use of surface cooling devices (blankets, pillows). Comparisons of these methods have not shown significant differences in terms of survival, neurological outcomes, and complications. However, intravenous cooling has demonstrated a lower risk of overcooling and rebound hyperthermia, as well as tighter temperature control, compared to other modalities of cooling [131,132,133,134].

The neuroprotective effects of hypothermia have been well documented in experimental models; their main pathophysiological mechanisms include reduced brain metabolism, anti-inflammatory action, inhibition of excitotoxic neurotransmitter release, and prevention of apoptosis. Clinical evidence was first described in the 1990s [135,136], followed by two pioneering trials, published in 2002, that demonstrated better neurological outcomes after induction of hypothermia in CA survivors when compared with normothermic control groups [137,138]. In a study published in 2019, a large trial included 584 patients who were randomized according to hypothermia (33 °C) or normothermia (37 °C). Despite not differing in mortality, the hypothermia group had a higher percentage of survivors with favorable neurologic outcomes on day 90 of evaluation [139,140]. Those studies did not show significant differences in adverse events between the groups, providing evidence that inducing hypothermia is safe. Importantly, both the duration of hypothermia and the ATM approach were different in these studies.

Regarding hypothermia timing, to date, only one clinical trial has compared the effect of ATM at 33 °C for 48 h versus 24 h on neurologic outcomes in unconscious PCA patients. This study included 355 patients in 10 intensive care units (ICUs) in six European countries. The authors found no differences in neurologic outcome or 6-month mortality, and adverse events were more likely to occur in the 48-h group (RR: 1.06; 95% CI: 1.01–1.1). Importantly, the authors emphasized that their study might have been insufficient to detect clinically important differences. As such, ATM remains a debatable issue [141]. Initially, the proposed temperature range, i.e., between 32 °C and 36 °C, was based on data from pioneering studies, which did not define a preferential temperature within this range. However, in 2013, a clinical trial with 939 patients from 36 ICUs in Europe and Australia compared the effects of inducing temperatures of 33 °C and 36 °C; the results showed no significant difference in neurological and mortality outcomes [142]. Due to the need for additional interventions to reach 33 °C and the significant side effects in this group, including arrhythmias with hemodynamic compromise, hyperlactatemia, and hyperglycemia, some authors have recommended the adoption of 36° as a target temperature [143,144,145,146,147]. In contrast, some studies after 2013 found that a limit of 36 °C achieved poor temperature control, with increased rates of fever and worse clinical outcomes [148,149,150,151].

A systematic review analyzed the main randomized controlled trials related to ATM, including 5509 patients for meta-analysis. Overall, patients treated at 33 °C showed similar rates of poor neurologic outcomes compared to those treated at 36 °C. However, when evaluating only studies that standardized the neurologic outcome by neurological scale score, patients treated at 33 °C showed a lower probability of poor neurologic outcome than those treated at 36 °C [152]. This analysis reinforces the importance of ATM as a relatively safe and effective strategy but also indicates that many questions about its effectiveness and implementation remain unanswered.

The largest and most recent trial on therapeutic hypothermia randomized 1850 patients to either induced therapeutic hypothermia at 33 °C versus normothermia, i.e., keeping the temperature below 37.8 °C. The results showed no benefit in the hypothermic group regardless of age, initial rhythm, or duration of resuscitation, and a higher incidence of arrhythmias with hemodynamic compromise. Despite that trial having the largest cohort on this topic and adequate methodology, we emphasize that the general management of PCA patients has improved over time. As such, in the current scenario, ATM appears to provide little statistical benefit. In addition, that study included only OHCA, evaluating a high incidence of CA secondary to coronary syndrome and a low occurrence of some events which are frequently seen in IHCA, such as shock. We note that even in the normothermic group, about 50% of the participants needed active ATM to avoid hyperthermia; thus, abandoning all temperature control protocols is ill-advised [153].

Some researchers maintain skepticism about the benefits of cooling and reiterate that the most important goal is to avoid hyperthermia [154], while others reinforce the idea that the use of poorly designed ATM protocols can cause unsatisfactory controls and fever escapes. Thus, trial results should be applied with caution in clinical practice, as an incorrect interpretation of the results can influence the outcome for patients. An ideal ATM approach based on patient characteristics remains undefined.

In summary, the European consensus advocates monitoring central temperature and the active prevention of fever (>37.7 °C) for at least 72 h after CA in patients who remain comatose, reinforcing the fact that there is not enough evidence to recommend for or against keeping the temperature between 32 °C and 36 °C [155].

Additionally, despite all the controversy, the most current literature cited in the International Liaison Committee on Resuscitation 2022 recommends actively preventing fever (with a target temperature equal to or less than 37.5 °C) in patients who remain comatose after ROSC. The recommended period of active fever prevention is at least 72 h in PCA patients who remain comatose. No temperature control techniques are preferential [156].

#### 5.1.2. Monitoring

Electroencephalographic monitoring is recommended for the early identification of seizure states and ideally should be applied continuously [153]. Abnormal electroencephalographic activity is observed in up to 30% of PCA patients and can be a cause or consequence of hypoxic-ischemic brain injury. The most common patterns include myoclonic and tonic-clonic seizures, with the former being more common and related to worse outcomes [154]. Fergusson et al. tested an invasive intracerebral catheter strategy to monitor pressure and cerebral tissue oxygenation compared with standard care without neuromonitoring. The invasive strategy was associated with better neurological outcomes. However, clinical trials are needed to confirm these findings [155].

Regarding interventions, crisis prophylaxis is not recommended. A recent trial that evaluated comatose survivors of CA did not show a benefit of suppressing rhythmic and periodic EEG activity with the use of antiseizure medication for at least 48 h plus standard care compared with standard care alone [157]. However, secondary prophylaxis is indicated with anticonvulsants, without specific drug predilection [48].

#### 5.1.3. Neuroprognosis

Neurological prognosis is an essential component of PCA care. Reliable assessments facilitate communication with family members and ensure therapeutic proportionality according to the patient’s wishes. In this context, the prognosis must be as specific as possible in order not to withdraw care prematurely in cases where patients may still recover. To this end, different data from clinical evaluations, serum markers, functional tests, and brain injury imaging methods are obtained [158].

Clinical examination should be performed to assess consciousness, reflexes, and myoclonus, i.e., the main parameters correlated with neuroprognoses. With this in mind, the poor accuracy of clinical findings, as well as confounding factors, such as medications, are important limitations to be considered. In a clinical scenario, scales are usually used to try to predict neurological outcomes. The most commonly applied scale after CA is Cerebral Performance Categories (CPCs). Alternatives to the CPCs include the modified Rankin Scale (mRS) and extended Glasgow Outcome Scale (GOSE). However, all of these scales have limitations and none was specifically designed to describe outcomes after global brain injury. Some authors have suggested dichotomizing neuroprognostication as ‘good’ or ‘poor’. Despite there being no consensus on what represents a poor neurological outcome, the latest version of the Utstein guidelines suggested that when dichotomizing neurological outcomes, a CPC value of 3–5 (or an mRS value of 4–6) is appropriate [158].

Regarding, biochemical data, blood biomarkers are presumed to correlate with the extent of lesions after CA. Neuron specific enolase (NSE), S-100B, and Tau are biomarkers which are released following injury to neurons, glial cells, and axons. Recently, microRNAs (miRNAs) have been identified as candidate biomarkers, although more investigations are needed to define protocols for their use. Electroencephalogram (EEG), in association with other predictors, can be used as a predictor of severity. The main patterns related to poor prognosis are status epilepticus or burst suppression after rewarming over an unreactive background. However, there is a lack of standardization of the different electrocardiographic patterns and their prognostic correlations. Short-latency somatosensory evoked potentials also can be used in neuroprognosis. The absence of the N_2_O wave is among the most robust predictors to be tested at 72 h after ROSC. Computerized tomography with cerebral edema and resonance magnetic hyperintense areas are the main imaging findings correlated with poor neurological outcomes in CA patients [158].

**Figure 2:**
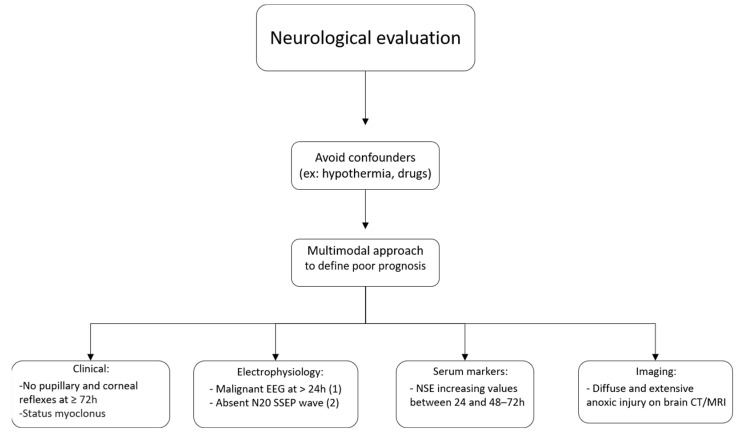
Neuroprognostication algorithm. Abbreviations: EEG—electroencephalogram, SSEP—somatosensory evoked potentials, NSE—neuron-specific enolase, CT—computerized tomography, MRI—Magnetic resonance Imaging. Legends: (1) suppressed background with or without periodic discharges and burst-suppression; (2) Bilateral absence of somatosensory evoked cortical N20-potentials.

None of the above modalities alone is able to predict the lack of neurological recovery with absolute certainty. Therefore, a multimodal approach should be applied in neurological assessments, and prognoses should be delayed whenever there is uncertainty. The current guidelines propose the use of algorithms to facilitate such multimodal assessments. Below, we present an adapted flowchart (Figure 2) that summarizes the main recommendations in the US and European cardiac arrest guidelines [48,105].

### 5.2. Hemodynamic Support

Treatment of PCA shock is similar to that for other types of shock, including optimizing preload, restoring perfusion pressure, and improving contractility to provide hemodynamic stability and prevent shock progression and organ failure. Resuscitation can be performed with fluids, vasopressors, inotropes, and/or transfusions. Norepinephrine should be considered a first-line vasopressor because it exhibits fewer arrhythmogenic side effects compared to other catecholamines [107]. Regarding inotropes, dobutamine is the most established treatment in this setting [159,160].

One of the goals of treatment is to stabilize the mean arterial pressure (MAP). Initially, observational studies suggested that higher MAP levels would promote better brain oxygenation [161] and neurological outcomes, increasing survival [162,163,164]. However, important randomized trials that compared a low normal MAP goal (between 65–75) to a high normal one (between 80–100) showed no differences in death, neurological outcomes, or even the required dosage of a brain injury marker such as neuron-specific enolase [165]. Reinforcing this fact, a significant recent publication also showed no difference in outcomes based on MAP targets (63 or 77 mmHg) [166].

However, it has recently been shown that targeting a high-normal MAP level was associated with lower troponin values, possibly corresponding to a lower degree of cardiac injury [167]. Thus, although it is currently recommended that hemodynamic treatments should be guided by blood pressure, the optimal MAP level is still unclear and may vary among patients [48]. Other variables that may help in managing hemodynamic support include urine output, lactate clearance, capillary refill time, and central venous oxygen saturation [168]. However, there is also a lack of targets for all these variables and a paucity of quality studies demonstrating the benefits of such strategies.

### 5.3. Coronary Reperfusion

Coronary angiography after CA is of potential diagnostic and therapeutic relevance when acute coronary occlusion is present. It is very important because the early revascularization of obstructed coronary arteries can reduce myocardial damage and its consequences, such as ventricular dysfunction, rhythm disturbances, heart failure, and death [169]. Evidence suggests that coronary artery disease (CAD) is present in up to 70% of patients resuscitated from OHCA (2–4), reinforcing the importance of this disease. However, it remains difficult to distinguish between acute coronary events and chronic CAD [170,171,172,173].

Baseline ECG is a basic instrument that helps identify the coronary event after resuscitation. It is important to note that ECG alterations are frequent in the early period after cardiac arrest. However, the persistence of ST-segment elevation after ROSC has a good positive predictive value (85%) in terms of identifying the presence of acute coronary injury [174]. Studies have identified the presence of coronary lesions in up to 80% of PCA patients with ST-elevation [175]. Observational studies have shown a survival benefit with early intervention; thus, the indication of early angiography in patients with STE is well established [176]. However, patients after CA were excluded from the main randomized trials that demonstrated the benefits of percutaneous coronary intervention in acute coronary syndromes. In patients without ST elevation, the real benefit of systematic angiography remains a matter of debate, given the cost and risks of the procedure. Initially, observational studies in patients without STE suggested better survival and neurologic outcome in the early (<24 h) angiography group compared to the late procedure or no procedure groups [177]. However, recently, three large randomized trials demonstrated a lack of benefit of early angiography in patients without ST elevation, even when analyzing rhythm-shockable patients [178,179,180]. The first study was the COACT, published in 2019. It included 552 patients without ST-elevation after resuscitation who were randomized into either immediate angiography or angiography after neurological recovery groups. No differences in survival outcomes were observed [178]. Another group published a trial in 2020 with 99 patients who were randomized into two groups: early angiography versus standard intensive care with the later angiography (or not); it also showed no difference in survival outcomes [179]. In 2021, a third trial evaluated the performance of immediate angiography compared with selected delayed angiography for patients without STE after out-of-hospital CA; it too confirmed the lack of superiority of one method over the other [180].

In addition to electrocardiography, studies are ongoing to identify other predictors of CAD in CA survivors to help determine which patients should undergo coronary angiography. One of these predictors is the elevation of cardiac troponin; however, despite its very high sensitivity, its low specificity makes it limited, from a clinical perspective, for the diagnosis of ACS in these conditions [181]. Other findings that may correlate with myocardial ischemia are segmental contractility abnormalities and left ventricular systolic dysfunction on transthoracic echocardiography. Although such findings are not specific and may result from coronary hypoperfusion and the applied resuscitation maneuvers, echocardiography is shown to be a complementary noninvasive option [182]. Therefore, despite all efforts, more evidence is needed to identify the characteristics of subgroups that would benefit from early coronary angiography, as opposed to those for whom the risk would outweigh the benefit.

Thus, despite the controversies, the main guidelines currently recommend that early angiography be performed for all patients with CA with suspected cardiac etiology and STE on ECG. For patients without STE with CA, it would be reasonable to consider emergency angiography in the presence of criteria such as electrical or hemodynamic instability. Although this subpopulation has not yet been evaluated in clinical trials, this early intervention may be beneficial [48,183].

### 5.4. Ventilatory Management

Most PCA patients are mechanically ventilated [184]. It seems interesting to consider protective ventilation in patients which are exposed to an intense inflammatory response, as it has been shown that ventilation strategies with lower tidal volumes (≤6 mL/kg) are independently associated with favorable neurocognitive outcomes, more days without ventilation, and more days without shock [185]. A sub-analysis of the Target Temperature Management-2 trial showed that protective ventilation is commonly applied in CA patients. The ventilator settings in the first 72 h after hospital admission, in particular, the driving pressure and respiratory rate, may influence six-month outcome. In addition, the authors suggested a formula ((4*driving pressure) + respiratory rate) that was independently associated with mortality and poor neurological outcomes [186].

Regarding blood gas indices, oxygenation and carbon dioxide disturbances may contribute to secondary brain injury [187]. Currently, the recommendation is to administer 100% oxygen until achieving oxygen saturation, and then to titrate the fraction of inspired oxygen to reach an oxygen saturation level above 92%, thereby ensuring sufficient supply to tissues while avoiding supraphysiological oxygen pressures, since hyperoxia can also be deleterious [129]. A recent randomized trial of various oxygen targets in comatose patients after CA found a similar incidence of death, severe disability, or coma with pO2 restrictive (68 to 75 mm Hg) and liberal (98 to 105 mm Hg) strategies [188]. It is recommended that carbon dioxide pressure values be maintained within a physiological range of 35–45 mmHg due to the deleterious effects on the CNS of hypocapnia, i.e., reducing blood flow, and hypercapnia, i.e., increased intracranial pressure [6].

### 5.5. Other Measures

Cardiac arrest centers: The implementation of cardiac arrest centers (CACs) is becoming increasingly widespread. A systematic review and meta-analysis that included more than 147,000 patients showed that CAC care was associated with improved survival and neurological outcomes in nontraumatic OHCA. Patients with shockable rhythms had greater benefits. However, the heterogeneity in CAC characteristics and types of patients transported to CACs underline that the associated literature should be interpreted with care [189].

Corticosteroids: There is currently insufficient evidence to support or refute the use of corticosteroids in PCA patients. Therefore, until there is greater certainty about their role, routine administration is not indicated [190].

Prevention of infections: The susceptibility to infection of PCA patients is increasing. Up to 65% of patients develop pneumonia [191]. In an attempt to reduce this outcome, some centers use prophylactic antibiotics; a retrospective study showed that this practice is associated with a reduction in pneumonia [192]. However, the same study showed no functional improvement or mortality reduction. This finding was reinforced by the results of a subsequent study with a large cohort [193]. In summary, the evidence supporting antibiotic prophylaxis is of limited quality, and as such, antibiotic prophylaxis is not recommended.

### 5.6. Future Perspectives

The future of post-arrest care is based on the discovery of new therapies which serve a clear purpose in PCAS, i.e., limiting its damage and helping the rehabilitation of survivors. The proposed therapies are based on the pathophysiology of PCAS. One of the first problems caused by IR in CA is energy depletion. In this context, a preclinical study of molar sodium lactate found that it was effective in limiting the severity of PCAS, proposing that high doses of this energy substrate may improve cardiac performance and brain function [194].

Another important contributor to PCAS is the systemic inflammatory response. Approaches to negatively modulate the inflammatory response have emerged as potential therapies. For example, the use of minocycline after ROSC has been associated with reduced brain levels of tumor necrosis factor-alpha, reduced neuronal death, and decreased activation of microglial cells [195]. Additionally, the use of sodium sulfide has been associated with improved neurological and myocardial function and reduced levels of cytokines. Both therapies have shown promise in animal models and are awaiting clinical validation. Another avenue of research is the use of microRNAs as anti-inflammatory regulators [196]. However, there is much to be learned about how to turn this approach into effective, patient-compatible, and targeted drugs [197].

Patients with PCAS have organ dysfunctions similar those observed in patients with sepsis. Thus, some authors have suggested that trials in patients with lung injury induced after CA may elucidate the potential of treatments traditionally used for sepsis. Cyclosporine is an immunomodulator that acts by preventing the opening of the mitochondrial permeability pore, one of the main mechanisms of cell injury that occurs during tissue reperfusion. Although its use has not shown benefits in terms of preventing multiple organ failures after resuscitation, later trials suggested that it may limit the severity of kidney damage 24 h after CA, serving as a possible protector drug [198,199].

One clinically tested measure is early use of hemodialysis to remove inflammatory mediators from plasma in order help circulatory recovery. A recent trial (HYPERDIA) randomized PCA patients but failed to demonstrate a difference with different times to ending use of vasopressors or levels of several important cytokines [200]. However, a prospective, multicenter, observational study with 1063 PCA patients found that renal replacement therapy associated with therapeutic hypothermia was associated with reduced mortality in the patients studied (KDIGO 3 PCA) [201]. Although it is an interesting proposal, additional research is needed to determine the merit of this measure.

Mechanical cardiac support may be considered in patients with refractory CA. Extracorporeal membrane oxygenation (ECMO) serves to reestablish circulation, maintaining organic perfusion despite the absence of a ROSC [202]. The challenge is to correctly identify candidates for such an expensive measure that is seldom available in practice. Bascom et al. proposed the use of a score, CREST, for the early identification of such patients [203]. This score assesses more than 25 parameters, including hemodynamic severity factors and neurological prognosis, to identify eligible patients. Another device that can help hemodynamic management is the intra-aortic balloon pump (IABP), as it can reduce cardiac work and oxygen demand, facilitating myocardial recovery. An evaluation of ECMO and IABP combination therapy in PCA patients was associated with improved survival with minimal neurologic impairment. When stratifying patients based on the underlying etiology of CA, only those with an ischemic etiology demonstrated better outcomes [204]. This finding had already been published in a meta-analysis that observed a benefit with this combination therapy in patients with CA secondary to ACS [204].

From a theoretical perspective, it is worth mentioning a model developed by ILCOR, published in 2019, which is intended to unify global assessments of post-cardiac patients. Such an approach was proposed to identify knowledge gaps and facilitate clinical research. However, it is difficult to evaluate studies with heterogeneous data resulting from, for example, different definitions of unfavorable neurological outcomes, the use of different scales to assess quality of life, and assessments of outcomes at different times (days, weeks, or even months) [205].

### 5.7. Rehabilitation

The success of CPR [206] in recent decades has increased the survival of patients who have suffered a CA [88]. Currently, about 11% of out-of-hospital CA patients [3] and 20% of IHCA patients survive hospital discharge [207,208]. However, the consequences of this catastrophic event include limitations in daily activities, participation in society, quality of life, and psychological condition [209]. Post-intensive-care syndrome encompasses the physical, psychological, and cognitive deficiencies which are common among survivors of critical illnesses [210]; CA can be a cause of this syndrome, which has shown increasing importance as a nosological entity.

Estimates of functional status after discharge of CA survivors show a wide variation, since the event produces heterogeneous pathological states, and relevant assessment instruments have not yet been standardized. Some registries show that 18% of out-of-hospital CA survivors and 40% of IHCA survivors had limited functional status at discharge [211]. Follow-up data indicate that fewer than half of these patients were able to return to work 6 months after hospital discharge. In addition, the main factors related to reduced social participation were cognitive impairment, depression, mobility problems, and fatigue [212]. The incidence of cognitive dysfunction one year after the event was estimated at 29%, while motor dependence was even more prevalent, affecting up to 43% of survivors [213]. It is also interesting to observe that Sandroni et al. showed that women are less likely than men to achieve a good quality of life after CA [214].

Health-related quality of life can be adequately assessed only when the patient is allowed to interact with their social environment. Therefore, it should be measured no earlier than three months post-CA. In this scenario, the Short-Form 36-Item Health Survey, the Health Utilities Index version 3, and the revised version of the EuroQol are currently recommended to assess quality of life after CA [214].

The rehabilitation of these patients represents the last part of the chain of survival; it is responsible for their reinsertion into society, with the help of the health system and caregivers. This is a complex task, because available evidence indicates that CA survivors undergo a long recovery period, requiring at least 11 weeks to develop a clinically relevant change in self-care skills [215]. We also reiterate that such planning should be carried out at an early stage, because a great part of the cognitive improvement occurs in the first 3 months of follow-up [216]. Additionally, caregivers may be affected by financial, social, or emotional problems [217,218], or they might get sick, making it even more difficult to ensure consistent care to patients after CA.

Therefore, it is imperative to understand the factors involved in the recovery of patients after CA; the absence of a coordinated plan to assess and reassess the survival of such patients hinders progress in this field. Despite the challenge of developing assessment models for patients with such a complex disease [219], health systems must find ways to share the knowledge they have obtained from their successes and failures so that improvements can be instituted.

Suggestions for a good rehabilitation plan include multidisciplinary outpatient follow-up such as physical, occupational, and speech therapy to help in recovery and adaptation to permanent disabilities. It is also important to provide a psychological and psychiatric follow up with an assessment of common conditions under these circumstances, such as anxiety, depression, and post-traumatic stress, both for CA survivors and their caregivers [48].

## 6. Conclusions

Improving survival after CA, with favorable neurological outcomes and good quality of life, is a challenging issue. Understanding the physiopathology of the condition is mandatory for the discovery of new treatment strategies and to obtain better results. Besides improvements in CPR maneuvers, the great increase in survival rates observed in recent decades have been due to post-cardiac arrest care. In this review, we have discussed the indications of ATM and early coronary angiography. In addition, rehabilitation plans are of fundamental importance and must be discussed with the patients and families.

## Figures and Tables

**Table 1:** Findings that may indicate CA etiology.

Organ System	Clinical Findings	Potential Etiology
Pulmonary	Diminished or abolished breath sounds	Unilateral: Pneumothorax, right mainstem intubation (left side less common) Bilateral: Pulmonary edema
Cardiovascular	New murmur	Papillary rupture, valve abnormality
	Unequal pulses or blood pressure	Aortic dissection
	Bradycardia	Toxidrome, hypoxia, hypo/hyperkalemia, hypo/hypercalcaemia, hypothermia, exogenous intoxication, acid-basic disturbances
	Tachycardia	Tachyarrhythmias, thyrotoxic storm
Abdomen	Distention/rigidity	Hemorrhage/inflammatory process
	Pulsatile mass	Aortic aneurism rupture
Extremity	Unilateral swelling/erythema	Pulmonary embolism, septic shock
	Hemodialysis fistula	Hyperkalemia
Skin	Cyanosis	Hypoxia, methemoglobinemia sulfhemoglobinemia
	Mottling, slow capillary refill	Septic shock, hemorrhagic shock
	IV injection sites	Drug overdose
	Open wounds/cellulitis	Septic shock
	Diffuse urticaria	Anaphylactic shock
Neurologic	Focal motor deficits	Stroke
	Global motor deficits	Toxidrome, hypoxic/anoxic brain injury

**Table 2:** Most important etiologies of cardiac arrest.

Organ System	Etiology
Cardiovascular	Acute coronary syndrome
	Arrhythmia
	Structural heart disease
	Cardiac tamponade
	Aortic dissection
Respiratory	Venous thromboembolism
	Upper airway obstruction
	Drowning
	Pneumothorax
	Hypoxia
Metabolic	Acidosis
	Hypo/Hyperkalemia
	Hypomagnesemia
Neurologic	Subarachnoid hemorrhage
	Status epilepticus
	Stroke
Others	Trauma
	Hypothermia
	Intoxication
	Non-traumatic exsanguination

## Data Availability

Not applicable.
